# Response to: Comment on “The Gut Microbiome Profile in Obesity: A Systematic Review”

**DOI:** 10.1155/2018/9109451

**Published:** 2018-12-20

**Authors:** Olga Castañer, Helmut Schröder

**Affiliations:** ^1^Cardiovascular Risk and Nutrition Research Group of IMIM-Hospital del Mar Research Institute, Barcelona, Spain; ^2^CIBER de Fisiopatología de la Obesidad y Nutrición (CIBEROBN), Santiago de Compostela, Spain; ^3^CIBER de Epidemiologia y Salud Pública (CIBERESP), Madrid, Spain

We appreciate the interest of Alwardat and colleagues [1] in our publication [2]. We agree with Alwardat et al. that the assessment of methodological quality for eligible studies is an important issue. Our review is aimed at addressing two topics as we stated at the end of the introduction, “This review focus on the current evidence of the associations between the microbiota profile and the individuals' phenotypes and on the effect of bariatric surgery on gut microbiota” [2].

The available data to address the first objective is limited to cross-sectional studies with the inherent limitation of this type of study design. The methodology of gut microbiota analysis differs among these studies. Although the most common technique used nowadays for characterizing microbial communities is 16S rRNA gene (rDNA) sequencing [3, 4], some of these studies have done targeted qPCR or DGGE (denaturing gradient gene electrophoresis). There is also a limitation produced by bias in metagenomic studies, in either the experiments or the data analyses [5]. The software used for high-throughput analyses is mostly QIIME software, and the specimen provided is most generally faecal samples, although we also included oral cavity or duodenal microbiota.

We found only four clinical trials that reported on the effect of bariatric surgery on gut microbiota. Additionally, the sample size of these trials was small, ranging from 6 to 21 participants. The main findings reflect that the microbial diversity increase after bariatric surgery depends mostly on the type of surgery. The methodological quality of these studies is comparable. However, due to the limited evidence, it is not possible to draw any firm conclusion on the effect of bariatric surgery on gut microbiota.

Egger's linear regression test and Begg's rank correlation test are statistical methods to detect publication bias in meta-analyses. However, these tests are not applicable in systematic reviews without quantitative effect size estimates.

We included a PRISMA flow diagram (Figure 1) in the first version of the manuscript. Following the suggestion of a reviewer, we deleted this diagram from the revised version of the manuscript.

It is correct that the PICO format (P: participants; I: intervention; C: comparison; O: outcomes) was not explicitly mentioned in our article, but it was clearly described in Tables 1 and 2 of our manuscript in the following columns: (1) description of the study, (2) population description, and (3) outcomes.

We have added a PRISMA flow diagram.

We acknowledge that the subtitle of Table 1 is incorrect. It should read, “Lean/obese microbiota cross-sectional studies”.

## Figures and Tables

**Figure 1:**
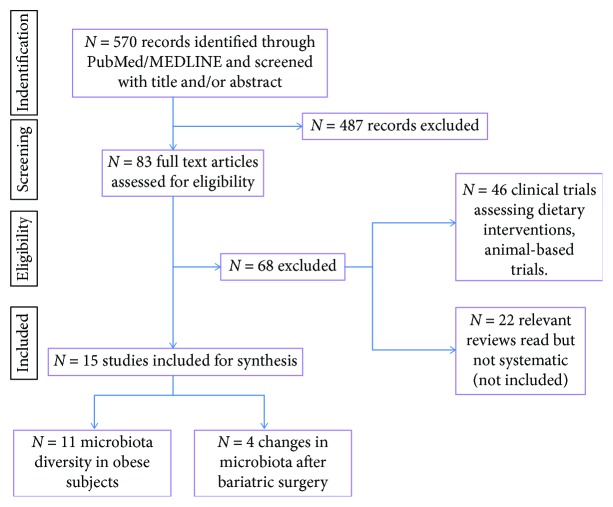
PRISMA flow diagram.
